# Automating surgical procedure extraction for society of surgeons adult cardiac surgery registry using pretrained language models

**DOI:** 10.1093/jamiaopen/ooae054

**Published:** 2024-07-24

**Authors:** Jaehyun Lee, Ishan Sharma, Nichole Arcaro, Eugene H Blackstone, A Marc Gillinov, Lars G Svensson, Tara Karamlou, David Chen

**Affiliations:** Cardiovascular Outcomes Research and Registries, Cleveland Clinic, Cleveland Clinic, Cleveland, OH 44195, United States; Cardiovascular Outcomes Research and Registries, Cleveland Clinic, Cleveland Clinic, Cleveland, OH 44195, United States; Cardiovascular Outcomes Research and Registries, Cleveland Clinic, Cleveland Clinic, Cleveland, OH 44195, United States; Cardiovascular Outcomes Research and Registries, Cleveland Clinic, Cleveland Clinic, Cleveland, OH 44195, United States; Heart, Vascular, and Thoracic Institute, Cleveland Clinic, Cleveland, OH 44195, United States; Quantitative Health Sciences, Lerner Research Institute, Cleveland Clinic, Cleveland, OH 44195, United States; Heart, Vascular, and Thoracic Institute, Cleveland Clinic, Cleveland, OH 44195, United States; Heart, Vascular, and Thoracic Institute, Cleveland Clinic, Cleveland, OH 44195, United States; Cardiovascular Outcomes Research and Registries, Cleveland Clinic, Cleveland Clinic, Cleveland, OH 44195, United States; Pediatric Institute, Cleveland Clinic, Cleveland, OH 44195, United States; Cardiovascular Outcomes Research and Registries, Cleveland Clinic, Cleveland Clinic, Cleveland, OH 44195, United States; Cardiovascular Innovation Research Center, Cleveland Clinic, Cleveland, OH 44195, United States

**Keywords:** artificial intelligence, clinical registries, cardiac surgery, natural language processing

## Abstract

**Objective:**

Surgical registries play a crucial role in clinical knowledge discovery, hospital quality assurance, and quality improvement. However, maintaining a surgical registry requires significant monetary and human resources given the wide gamut of information abstracted from medical records ranging from patient co-morbidities to procedural details to post-operative outcomes. Although natural language processing (NLP) methods such as pretrained language models (PLMs) have promised automation of this process, there are yet substantial barriers to implementation. In particular, constant shifts in both underlying data and required registry content are hurdles to the application of NLP technologies.

**Materials and Methods:**

In our work, we evaluate the application of PLMs for automating the population of the Society of Thoracic Surgeons (STSs) adult cardiac surgery registry (ACS) procedural elements, for which we term Cardiovascular Surgery Bidirectional Encoder Representations from Transformers (CS-BERT). CS-BERT was validated across multiple satellite sites and versions of the STS-ACS registry.

**Results:**

CS-BERT performed well (F1 score of 0.8417 ± 0.1838) in common cardiac surgery procedures compared to models based on diagnosis codes (F1 score of 0.6130 ± 0.0010). The model also generalized well to satellite sites and across different versions of the STS-ACS registry.

**Discussion and Conclusions:**

This study provides evidence that PLMs can be used to extract the more common cardiac surgery procedure variables in the STS-ACS registry, potentially reducing need for expensive human annotation and wide scale dissemination. Further research is needed for rare procedural variables which suffer from both lack of data and variable documentation quality.

## Introduction

The Society of Thoracic Surgeons (STS) Adult Cardiac Surgery Database (STS-ACSD)^1^ was established in 1989 to provide a nationally agreed-upon database for adult cardiac surgeries to identify “best practices and potential gaps, and evaluate their performance against national and regional competitors.” Initially focused on coronary artery bypass grafting (CABG), the STS-ACSD now includes a wide range of aortic valve replacement/repair (AVRR) and mitral valve replacement/repair (MVRR) procedures among other cardiac procedures. The registry plays an important role in public transparency and accountability related to surgical practice. The registry also supports clinical discovery of best surgical practices informed by the over 7.5 million patient records.^2^ National registries are a powerful source of comprehensive and detailed information complementary to electronic health record (EHR) data, which are often populated for the purpose of reimbursement rather than clinical discovery.^3^^,^^4^

The quality and integrity of the data is supported by labor-intensive manual abstraction which is time-consuming and error-prone.^5^ The registry itself contains over 300 variables associated with each procedure including demographics, comorbidities, laboratory results, imaging findings, hemodynamics, details on surgical techniques used, and in-hospital (or 30-day) outcomes that are required to be manually abstracted.^6^ Such information, particularly granular elements such as type of aortic valve repair are often not easily found within the structured portion of the EHR. Therefore, hospitals with high procedural volume participating in the registry must employ a significant number of personnel to achieve the data quality and completeness standards.^7^ As healthcare systems grow, there will be greater pressure on this inherently unscalable resource.

Natural language processing (NLP) techniques have been proposed to provide high throughput data extraction from free-text sources at scale. Rule based methods which combined curated dictionaries of terms and pre-defined sentence patterns in order to identify clinical concepts^8^ have been used in highly targeted tasks in surgical applications such as to identify adverse outcomes including surgical site infection,^9^ bleeding,^10^ acute kidney injury,^11^ among others. They have also been applied to more general task of information extraction to build research registries,^12^ although require significant investment of time and expertise to engineer them for each new application.

Pretrained language models (PLMs) have produced huge leaps in performance in general NLP tasks such as reading comprehension, question answering tasks, and neural translation by implicitly modeling patterns and relationships in text. Thereby, it has widely been suggested that PLMs can enable scalable information extraction for quality and research.^13^ Kim et al leveraged such models to generate billing codes directly from text.^14^ Similarly, Burns et al used a deep learning architecture to generate anesthesiology Current Procedural Terminology (CPT) codes from free text notes.^15^ However, to our knowledge, no work rigorously developed and validated a model to automate the abstraction of a national surgical registry using NLP.

In this article, we present a PLM fine-tuned on a large corpus of surgical notes aimed at automating the extraction of surgical procedural data required for the largest national cardiac surgery registry at a level of accuracy at or above what is demanded by the STS. The proposed model, cardiac surgery bidirectional encoder representation from transformers (CS-BERT) is trained using only data from free text operative notes and records from Cleveland Clinic main campus. However, the model was able to generalize to satellite campuses with significantly different case complexity, case mix, and documentation standards. We demonstrate that CS-BERT achieves near human level performance on many registry variables, suggesting its possible use for both research and operational tasks.

## Methods

### Data

The Cleveland Clinic Institutional Review Board waived the need to obtain informed consent for the purposes of this work (IRB #23-1232). The training and test cohort of this work came from patients in our STS-ACSD registry between 2011 and 2023. The inclusion criteria for this registry were any patient who was undergoing CABG, aortic valve (AV) replacement or repair, or mitral valve (MV) replacement or repair. The registry was first started in 1989, but we included only the years which operative reports were available in our her. Outside users of the registry can query the national registry for aggregate data for clinical discovery research while users internal to our institution with the appropriate training and IRB have access to patient level information.

### Registry preprocessing

One of the difficulties with clinical registries is the evolving versions over time representing both changing semantics and clinical practice.^16^^,^^17^ For the latter, our institution has created a shared data model to map different versions of the registry into a single dataset. Details of the data model is included in Table S1. As an example, only replacements and resections of pulmonic valves were tracked in version 2.61. The latest version adds repairs and thrombus removal to this variable. Furthermore, the common mapping includes rules based on the hierarchical configuration of the data elements to fill in certain “NA’s.” For example, if a subcategory of AV repair is selected (eg, ring annuloplasty), then AV repair is automatically selected as well. We then impute any remaining “NA’s” as “No’s.” The SQL code for this mapping is included in the public code repository.

The current 4.20 version of the STS ACS registry consists of over 400 elements, although this number changes with the version of the registry. A large number of them are completely empty due to changes in surgical practice over the years. Therefore, we trained the model to identify only elements with non-zero occurrence in the latest 4.20 version of the STS-ACS registry, leaving 94 variables.

### Note preprocessing

**Figure 1.**
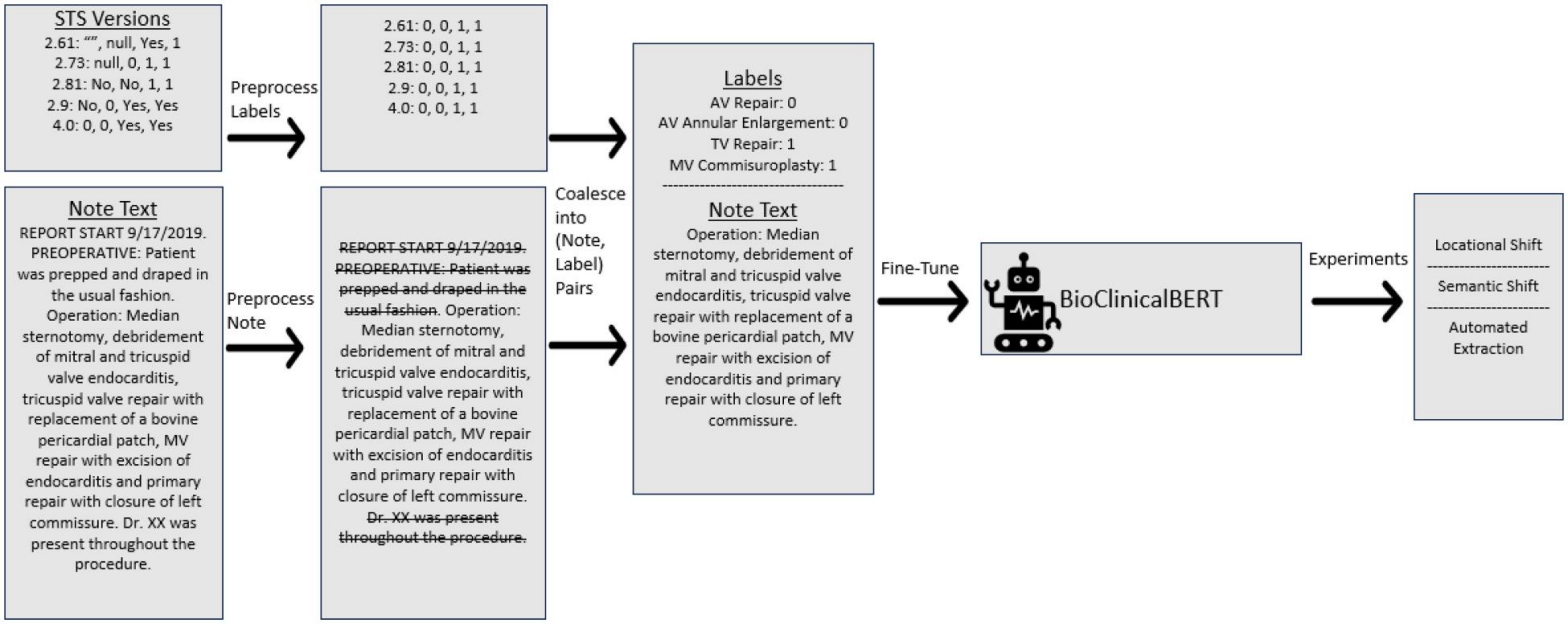
General overview of data preparation, fine-tuning, and study design for validation of CS-BERT.

We standardized text by removing content that is not relevant to the surgery from the operative report including address, physician, and patient names. These sections were removed using a rule-based method which identified section headings. We focus on the indications, procedure, findings, and anesthesia sections of the note. All non-informative sections were removed using a rule-based method which identified section headings. Sections spanned the start of a heading to the start of the next found heading title. Next, the text was divided into sentences using a clinical sentence segmentation tool from MedSpaCy.^18^ The preprocessing step for extracting relevant sections and cleaning the multilabels are shown in Figure 1. Therefore, redundant operative information in the conclusion, irrelevantly autogenerated headers, and names are removed from the notes.

### Model

We base our model on BioClinicalBERT,^19^ a clinical domain specific version of BERT.^20^ A linear probe with a sigmoid activation layer was added following the last layer of the BioClinicalBERT model to perform multi-label classification of the whole operative report (shown in Figure 1). We take a classification approach instead of a named entity recognition approach as operative details are often not explicitly written in the note. For example, aortic ring annuloplasty is a common AV repair technique but often not specifically written in the note as it is part of another procedure such as bicuspid repair.

### Training

We fine-tuned a multi-label classifier for binary surgical labels for each operative report using the previously described BERT architecture. We evaluated several hyperparameters including the number of layers to fine-tune, learning rate, and batch size. For optimization, we employed the AdamW^21^ algorithm with an exponential learning rate scheduler that gradually reduced the learning rate with a decay factor of *γ* = 0.98 at every third epoch, and binary cross entropy loss. The hyperparameters were selected from Bayesian optimization using the Tree-structured Parzen Estimator (TPE).^22^ The final hyperparameters used for training were a learning rate of 1 × 10^−3^, batch size of 256, and updating only the final transformer block in the pretrained BERT model.

### Experiments

We validate the generalizability of CS-BERT in several experiments. We split this dataset by campus sites and different versions of the STS-ACSD to prevent data leakage during testing. We trained the model using only main campus data and STS-ACSD versions 2.61–2.90. We kept the most recent STS-ACSD version 4.20, as well as data from other institutions as testing cohorts. If a patient had multiple surgeries, only their first surgery was used to prevent potential data leakage to other sites or versions of the registry. Our primary evaluation metrics encompassed macro-averaged area under Precision-Recall curve (AUPRC) for data imbalance issues, precision, recall, and the F1 score. We calculate confidence interval using bootstrapping. We also break up aggregate results into common and long-tail variables, with the long tail being defined as any STS-ACS variable that occurred in less than 5% of cases. This is done because highly unbalanced data is difficult to learn, and our status as an academic quaternary care center makes rare procedures to be more likely and not applicable to many hospital systems.

First, we evaluated CS-BERT’s performance on an in-context test set of Main Campus surgical events. CS-BERT was also compared against a random forest (RF) model trained using only CPT codes associated with each surgery as recorded in our EHR to evaluate the need for free-text data to populate STS-ACS variables. These codes were pivoted into a one-hot vector for each surgical encounter to match the same cohort used for NLP development. The RF model was trained with sklearn’s default configuration.^23^ We further took an in-depth analysis of where the models fail to better understand both the model and the underlying documentation quality.

We further conducted experiments on the generalizability of CS-BERT to unseen data and for sensitivity to semantic shifts over time in data definitions as depicted in the last block of Figure 1. As stated earlier, a single STS-ACSD may subtly change definitions over different STS versions. Therefore, we train a model with data outside of a specific version (eg, for v4.20, we train with v2.61-2.91). This is a natural experiment given that each version covers different periods in time and represents subtle shifts in data definitions. For generalizability to unseen sites, we trained CS-BERT using only Main Campus data and evaluated on satellite sites (Fairview and Hillcrest). The model’s performance was only evaluated on “common procedural variables” given the relative rarity of the long-tail procedures in each of these test sets.

We evaluated feasibility of using our model for outcomes research. We compared the rate of adverse events/outcomes (30-day survival, readmission, and permanent stroke) for AV replacement and repair, MV replacement and repair, and CABG using the manually abstracted STS ACS data and the predicted output of our model. The statistical difference between outcomes of procedures ascertained by humans and by CS-BERT was compared using paired *t*-test. We further asked the question if less data may accentuate errors between human and the model by undersampling the data and building a model from the undersampled data.

## Results

We trained and validated this model on procedures captured by STS-ACS that were performed within the Cleveland Clinic Health Systems between 2011 and 2023 summarized in Table 1. We retrieved the 85 922 operative reports associated with these procedures from our EHR and generalized the dataset as a 1-to-1 relationship between a surgical event to surgical note, where a surgical event is unique to the patient id, surgery date, and note id tuple. Following the removal of non-relevant sections, the average number of words in each operative report was reduced from 289.9 ± 228.3 to 170.0 ± 128.3. The total number of tokens in our training data is approximately 35 million.

**Table 1.** Breakdown of caseload and demographics of cardiovascular surgery patient cohort within each site.

	Main campus	Hillcrest	Fairview
**Sex**			
Male	37 478 (66.6%)	9721 (66.1%)	10 003 (66.7%)
Female	18 763 (33.3%)	4986 (33.9%)	5001 (33.3%)
**Ethnicity**			
Asian	817 (1.5%)	169 (1.1%)	150 (1.0%)
Black	3707 (6.6%)	1867 (12.7%)	1573 (10.7%)
Others	2721 (4.8%)	534 (3.6%)	742 (4.9%)
White	48 996 (87.1%)	12 137 (82.5%)	12 539 (85.6%)
**Hispanic**			
Yes	1289 (5.4%)	256 (1.7%)	383 (2.6%)
**Comorbidities**			
Diabetes	14 985 (26.6%)	5033 (34.2%)	5046 (34.5%)
Hypertension	43 529 (77.4%)	11 955 (81.3%)	12 044 (82.3%)
Stroke	5463 (9.7%)	1604 (10.9%)	1698 (11.6%)
Heart failure	17 388 (30.9%)	4559 (31.0%)	4869 (33.3%)
Chronic lung disease	13 603 (24.2%)	3741 (25.4%)	4048 (27.0%)
Family CAD^a^	7743 (13.8%)	1957 (13.3%)	2031 (13.5%)
Smoking	27 781 (49.4%)	7590 (51.6%)	7911 (52.7%)
**Procedures**			
Length of surgery (min)	294.2 ± 122.8	292.9 ± 121.5	291.2 ± 121.8
AV replacement^b^	20 096 (35.7%)	4652 (31.6%)	4866 (33.1%)
AV repair^c^	2716 (4.8%)	534 (3.6%)	545 (3.6%)
CABG^d^	19 738 (35.1%)	6922 (47.1%)	6867 (45.8%)
MV replacement^e^	5370 (9.6%)	1126 (7.7%)	1172 (7.8%)
**Note statistics**			
Positive per surgical event	8.20 ± 3.35	7.50 ± 3.35	7.51 ± 3.35
Words in note	579.7 ± 325.9	600.6 ± 322.3	585.2 ± 297.5
Words in procedure section	170.8 ± 138.0	185.4 ± 160.0	174.8 ± 140.0

Length of surgery is defined as time of first incision to time of closing in minutes.

a Coronary artery disease

b Aortic valve

c Aortic valve

d Coronary artery bypass graft

e Mitral valve

### CS-BERT accurately identifies surgical procedures captured in the STS ACS registry from operative notes

We show aggregate CS-BERT results in Table 2 and results broken down into specific variables in Table S2. The classifier is capable of accurately identifying broadest categories of procedures, such as CABG (F1 0.9526), AV replacement (F1 0.9468), MV replacement (F1 0.9502), and MV repair surgeries (F1 0.9417) using only the free-text operative notes as inputs. One variable that stands out is AV repairs (F1 0.6218), which results lag significantly. The macro-averaged performance across all procedural concepts is 0.4650 ± 0.3389 in F1 and 0.4556 ± 0.3592 in AUPRC. However, out of the 94 variables, 21 variables reached over 0.9 of AUPRC. Much of this result is likely due to the relative imbalance of many variables, with 58 of the variables having less than 5% positive instances.

**Table 2.** Performance difference between common (variables with >5% occurrence) and those in the long tail surgical labels in macro-statistics.

	F1	AUPRC	Precision	Recall
All	0.4554 ± 0.3389	0.4556 ± 0.3592	0.4621 ± 0.3388	0.4699 ± 0.3387
Common	0.8417 ± 0.1838	0.8670 ± 0.1872	0.8403 ± 0.1845	0.8444 ± 0.1832
Long-tail	0.2970 ± 0.2432	0.2720 ± 0.2475	0.2934 ± 0.2412	0.3028 ± 0.2450

Table 3 provide examples of the predicted results of false positives and negatives. In some, a false positive should have considered a true positive due to an error in the label as with the AV repair which should have been labeled an AV replacement. A false positive case of AV repair addresses a case of an interrupted suture with pledgeted sutures, which is a common suture technique in AV replacement. On the other hand, the false negative case of AV repair gets confused with the construction of Carpentier-Edwards valve leading to predicting AV replacement. Alternatively, the model does sometimes have difficulty with mixed context where a repair could have been done on one valve (MV valve in the example) and a replacement in another (AV valve). The false positive case of AV annular enlargement mentions MV repair, which possibly confuses the model, whereas the false negative case consisting of numerous implant types and lack of keywords of AV annular enlargement possibly led confuse the model to miss AV annular enlargement. Finally, some notes just did not contain enough information, such as the false negative AV replacement. In this case, an unfinished note was pulled instead of the final, thereby leaving important information out.

**Table 3.** Examples of when the model failed to correctly identify certain features.

Surgery	False positive	False negative
AV repair	An aortotomy was performed. The aortic valve leaflets were excised. The annulus was debrided. The left ventricle and aortic root were then copiously irrigated to remove any debris. A *21-mm St. Jude tricuspid aortic valve was sutured in place* with pledgeted 2-0 sutures. The pledgets were placed on the ventricular side and the valve seated well. The aortotomy was closed in 2 layers first with a running horizontal 4-0 Prolene, then a running over-and-over 4-0 Prolene.	First, we did aortotomy at the anastomosis between the autograft and the aorta. We inspected the autograft valve, and the pathology was found as described. We did put 2 commissuroplasty sutures; 1 around each of the commissures on either side of the left coronary cusp. The aortotomy was closed with a running 4-0 Prolene. Next, *the old homograft was carefully peeled out* trying to be reasonably radical. There was a lot of scar tissue towards the aorta, and we did not release the takeoff of the right pulmonary artery. The right ventricular *outflow tract was eventually reconstructed with a 30-mm Carpentier-Edwards valve conduit including a 31-mm porcine valve*. The conduit was sutured in both ends with a running 4-0 Prolene.
AV annular enlargement	A bulldog clamp was placed on the patent ITA graft during the cross-clamp period. A length of saphenous vein graft was used to bypass the posterior descending artery. A second length of saphenous vein graft was used to bypass the ramus intermedius and a third length of saphenous vein graft was used as a sequential graft first with a side-to-side anastomosis to the first diagonal and then an end-to-side anastomosis to the second diagonal. All 4 distal anastomoses were constructed with running 7-0 Prolene suture and prior to completing the anastomoses, they were probed and found to be widely patent. A left atriotomy was performed and the mitral valve was repaired with a 28-mm Medtronic Profile 3D ring. This was sutured in place with interrupted 2-0 Ethibond sutures. At the completion of the repair, the valve was tested and appeared to be competent. The left atriotomy was closed with a running 3-0 Prolene.	The aorta was opened with an oblique aortotomy. It was clear that this was a subcoronary homograft. The valve itself is calcified and that accounts for some of the aortic stenosis but in addition to that there is tissue ingrowth creating a small orifice actually beneath the valve. With a *#15 blade we carved both the subvalvular and the valve out, removing most of the previously placed homograft*, in fact, endarterectomizing the area of the non-coronary cusp in order to get the valve out. It still looked pretty small so at this point we took a tear drop-shaped Periguard patch and sewed it into place so that we could implant a #21 bovine pericardial valve where there had been a #19 homograft previously. The valve was inserted with horizontal mattress sutures of 2-0 Tycron backed with Teflon felt and in the area of the non-coronary cusp where the Periguard was the sutures were placed inside out on the graft and tied down over a strip of felt. The Periguard then was used to close the aorta and we used 4-0 Prolene for this.
AV replacement	Carbon dioxide was flooded over the operative field and the ascending aortic aneurysm opened vertically up to the junction of the tubular ascending aorta with the transverse aortic arch. There, a 5 mm cough was maintained and the aorta transected completely. A 30-mm Hemashield graft was selected for implantation and an end-to-end anastomosis created from the *Hemashield graft to the distal tubular ascending aorta* utilizing a running 3-0 Prolene suture. The anastomosis was then tested and found to be hemostatic. t to the base of the aortic root. Visual inspection of the Carbomedics prosthesis showed it to be completely intact. The leaflets open and close appropriately.	The patient was taken to Cath Lab and prepped and draped in the usual manner, and the vascular sheath and wires were inserted the groins and fed up into the left ventricle. *Percutaneous aortic valve insertion.*

Italic areas highlight sections of text indicating likely mismatch. False positive refers to when the model predicts the procedure involves a certain procedural variable but does not. False negative refers to when the model misses out on a variable that should have been captured.

### Deep learning approach enables more detailed extraction of data compared to relying on the EHR

**Figure 2.**
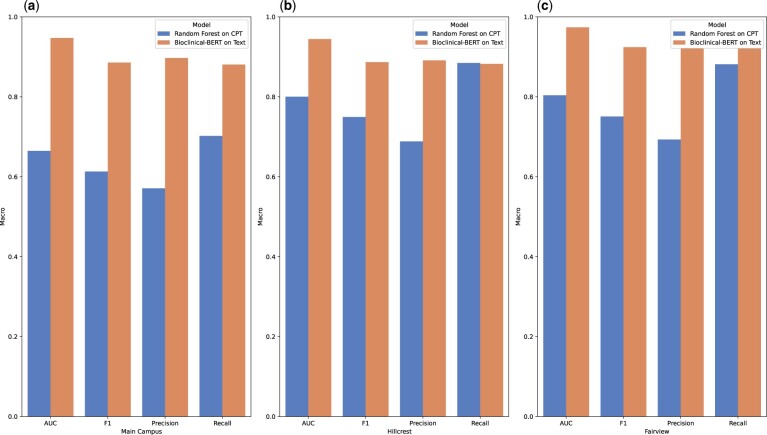
The model’s macro averaged F1 score compared with CPT codes on (a) Main Campus, (b) Hillcrest, and (c) Fairview.

Our CS-BERT model outperforms predictions based on CPT codes as depicted in Figure 2, where our model (F1 of 0.8819, 0.8832, 0.8822) clearly outperforms the CPT-based RF model (F1 of 0.6121, 0.6102, 0.6163) across all 3 campuses. This is unsurprising given that billing does not require the same level of granularity as medical registries (and for many reasons specifically discourages high granularity).^24^ However, there are a subset of procedure variables which have similar granularity to specific CPT codes (eg, AV replacement) where accuracy is similar for both CPT and CS-BERT. This would suggest that our model has near human level competence given CPT codes are also human coded.^25^ Furthermore, the overall accuracy of the model is at or above the 5% error rate that is mandated by the STS.^26^

### CS-BERT generalizes to different sites

One of the main barriers of wide implementation of deep learning NLP models is the variation in clinical note content and style across different institutions. Heterogeneity of narratives across different institutions may be problematic when the provenance of data is confined to a small number of institutions that are not representative.^27^ In consideration of subsequent overfitting, it is imperative to test generalizability over a wide range of clinical narratives on the classifier. We applied the model trained on main campus data and applied it to different hospital sites without finetuning. We found our model did generalize well to unseen clinical notes in other institutions within our health systems with no significant difference in AUC or F1 score across institutions as shown in Table 4. Although there is increasing push to centralize and standardize certain practices across different institutions, the individual culture and practices of our sites are relatively independent. Both these satellite hospitals have much higher number of CABG procedures compared to main campus and much fewer AV repairs and MV replacements. Furthermore, there are certain surgeons who only work in the satellite sites. Therefore, the quality of notes and the diversity of standard surgical practice across different sites is still quite large.

**Table 4.** Measure of generalizability to other hospital system of common variables.

Sites	F1	AUC	Precision	Recall
All sites	0.8887 ± 0.0102	0.9474 ± 0.0069	0.8927 ± 0.0082	0.8857 ± 0.0122
Main campus	0.8856 ± 0.0077	0.9473 ± 0.0056	0.8972 ± 0.0082	0.8806 ± 0.0085
Fairview	0.9243 ± 0.0122	0.9738 ± 0.0081	0.9253 ± 0.0122	0.9238 ± 0.0123
Hillcrest	0.8867 ± 0.0127	0.9444 ± 0.0076	0.8911 ± 0.0118	0.8824 ± 0.0136

### CS-BERT is robust to semantic shift

Another important aspect about the robustness of deep learning models for registry abstraction is the constant semantic shift over time. Robustness to semantic shift means no degradation in performance due to differences in lexical deformations and diachronic changes. As standard surgical practices change with better clinical knowledge, the content of the registries also change. We show that our method is more robust to such changes with minimum amount of retraining necessary in Table 4. Each row in the table contains an STS version that is trained on every version besides the version specified in the STS Version column from main campus. Given that our model is trained using data from prior versions, which is the case of the last row of Table 4, the generalizability to new data is quite robust. Although the model’s performance did not degrade substantially with new registry versions, the model did fail to generalize backwards in time. We believe this is due to substantial differences in practice such as the introduction of transcatheter aortic valve replacement (TAVR) in 2014 and the separation of aortic root procedures from aortic valve procedures in version 2.73 from 2.61.^2^

### Automated extraction using CS-BERT provides similar outcomes compared to manually abstracted registry data

Given the main purpose of STS-ACS and other clinical registries are outcomes research and reporting, it is important to ensure that an automated system reports the same outcome metrics. We evaluate the aggregate 30-day mortality, readmission, and permanent stroke of AV replacement and repair, MV replacement and repair, and CABG using both CS-BERT extracted procedures and manually extracted procedures recorded in the STS-ACS. We show that there are no clinically significant differences in adverse outcomes using as little as 10% of the data in Table 5. This provides additional evidence that CS-BERT can potentially be used to augment manual annotation.

**Table 5.** Cross-validation of CS-BERT divided by STS-ACS versions.

STS version	F1	AUPRC	Precision	Recall
2.61	0.8332 ± 0.0212	0.6319 ± 0.0221	0.8356 ± 0.0224	0.8309 ± 0.0202
2.73	0.8864 ± 0.0472	0.9658 ± 0.0178	0.8941 ± 0.0745	0.8894 ± 0.0075
2.81	0.8833 ± 0.0302	0.9733 ± 0.0113	0.8866 ± 0.0516	0.8865 ± 0.0116
2.9	0.8695 ± 0.0527	0.9688 ± 0.0112	0.8753 ± 0.0908	0.8866 ± 0.0114
4.2	0.8856 ± 0.0208	0.9845 ± 0.0033	0.8924 ± 0.0206	0.8801 ± 0.0122

The model is fairly stable except for the earliest version of the registry.

## Discussion

The maintenance of clinical registries is a critical but expensive piece of a trustworthy healthcare system. The cost of maintenance can be prohibitive to small and medium-sized healthcare facilities due to the additional human resources demands, therefore limiting transparency. Automated abstraction has the potential to minimize clerical work and improve clinical oversight, although the technology is yet unproven. This work provides evidence that some of the most common parts of this task can be reliably automated through NLP models, thereby reducing clerical burden and improving the breadth of outcomes-based research. Most importantly, we demonstrate that surgical procedures from our model produces similar outcomes as our manual annotated data (Table 5), providing evidence that outcome reporting can be automated.

Our work represents a significant innovation of breadth of variables and validation rigor for surgical data extraction. Prior work in this area have been primarily focused on extracting adverse outcomes such as post-operative bleeding^10^ or surgical site infection^9^ in relation to surgical registries. For instance, Shen et al developed dictionaries of key words in progress notes to identify surgical site infection.^28^ Although adverse outcomes are an important aspect of registries, they are only a small portion of the required data such as granular procedural details (eg, repair type). Alternatively, there have been significant effort to automate clinical billing. Automating billing requires a much broader approach towards note classification given the broad set of possible billing codes generated from a procedural note. Burns et al proposed a similar model training method to develop a model to predict over 250 unique CPT codes from operative notes, achieving 88% accuracy.^15^ However, the STS ACSD contains more granular information as evident that the poor performance of the RF model trained using just CPT codes. Furthermore, we have shown more rigorous evaluation of our model through generation to different sites and potential semantic shift over time.

This model addresses several barriers facing registry data extraction. First, differences in documentation standards and practice can impact NLP information extraction models.^27^ There is less likely to be practice variations in documentation standards given the single data source (operative reports) between institutions. However, both syntactic and semantic changes^29^ can still impact NLP efficacy. We show that despite differences in procedural lengths and distributions, CS-BERT generalizes well to these satellite sites (Table 6).

**Table 6.** Comparison of aggregate surgical outcomes in procedures ascertained manually or CS-BERT.

Variable	% of data	30 day survival (%)		Readmit (%)		Stroke permanent (%)	
		STS-ACSD	CS-BERT	STS-ACSD	CS-BERT	STS-ACSD	CS-BERT
AV replacement	100%	0.0157 ± <0.0001	0.015472 ± <0.0001	0.0293 ± <0.0001	0.0305 ± <0.0001	0.0212 ± <0.0001	0.0209 ± <0.0001
	50%	0.0160 ± 0.0017	0.0156 ± 0.0017	0.0292 ± 0.0024	0.0306 ± 0.0025	0.0213 ± 0.0018	0.0210 ± 0.0017
	10%	0.0149 ± 0.0071	0.0144 ± 0.0064	0.0305 ± 0.0077	0.0316 ± 0.0067	0.0223 ± 0.0063	0.0219 ± 0.0060
AV repair	100%	0.0081 ± <0.0001	0.0130 ± <0.0001	0.0404 ± <0.0001	0.0346 ± <0.0001	0.0242 ± <0.0001	0.0194 ± <0.0001
	50%	0.0078 ± 0.0051	0.0140 ± 0.0058	0.0389 ± 0.0111	0.0342 ± 0.0102	0.0242 ± 0.0074	0.0195 ± 0.0059
	10%	0.0050 ± 0.0093	0.0112 ± 0.0160	0.0347 ± 0.0229	0.0350 ± 0.0236	0.0213 ± 0.0233	0.0192 ± 0.0202
MV replacement	100%	0.0116 ± <0.0001	0.0112 ± <0.0001	0.0341 ± <0.0001	0.0337 ± <0.0001	0.0163 ± <0.0001	0.0160 ± <0.0001
	50%	0.0120 ± 0.0029	0.0118 ± 0.0034	0.0347 ± 0.0054	0.0342 ± 0.0059	0.0152 ± 0.0037	0.0151 ± 0.0036
	10%	0.0130 ± 0.0092	0.0113 ± 0.0090	0.0381 ± 0.0146	0.0383 ± 0.0145	0.0164 ± 0.0104	0.0159 ± 0.0110
MV repair	100%	0.0119 ± <0.0001	0.0124 ± <0.0001	0.0334 ± 0.0001	0.0362 ± 0.0001	0.0203 ± <0.0001	0.0207 ± <0.0001
	50%	0.0113 ± 0.0023	0.0117 ± 0.0026	0.0339 ± 0.0039	0.0367 ± 0.0041	0.0203 ± 0.0027	0.0209 ± 0.0027
	10%	0.0096 ± 0.0060	0.0101 ± 0.0056	0.0348 ± 0.0142	0.0374 ± 0.0146	0.0221 ± 0.0070	0.0225 ± 0.0066
CABG	100%	0.0143 ± <0.0001	0.0148 ± <0.0001	0.0306 ± <0.0001	0.0316 ± <0.0001	0.0201 ± <0.0001	0.0203 ± <0.0001
	50%	0.0143 ± 0.0010	0.0148 ± 0.0011	0.0302 ± 0.0028	0.0310 ± 0.0028	0.0192 ± 0.0019	0.0196 ± 0.0019
	10%	0.0138 ± 0.0045	0.0142 ± 0.0047	0.0290 ± 0.0072	0.0303 ± 0.0068	0.0193 ± 0.0053	0.0194 ± 0.0056

The data percentage refers to random sub-sampling to measure the impact of lower volume on potential errors in aggregate outcomes. There are no cases where outcomes from CS-BERT is statistically different from manually abstracted procedures.

Second, registries evolve over time with changes priorities and new development in healthcare (eg, introduction of TAVR). Such changes can impact the efficacy of automated data extraction and potentially require expensive re-training of the model. The STS-ACSD represents such changes with new revisions coming every 3-5 years. We demonstrate that our model is robust against these changes given the relatively stable data extraction across versions (Table 4). This is important given that most sites do not have the computational capabilities, nor the surgical volume to support fine-tuning the model with regular new updates to the semantics and content extracted by the registry.

There are 2 major problems with relying on registries and only operative notes for data extraction. As with any data source, we are limited to the accuracy of human annotators. Although the STS does audit centers, there is still an appreciable level of error (<5% error is expected^2^). This also varies for different variables as the STS audits are concentrated on the high-level surgical category (eg, AV replacement and repair, MV replacement and repair, and CABG) and adverse outcomes. Second, the completeness of the actual operative reports is variable. As shown in Table 3, many reports do not include granular details of the procedure. Further, the trends towards templated operative notes will standardize what is collected, but the included details are dependent on current coding and clinical knowledge. New details about procedures or diseases that might be otherwise be written down may be lost due to the templated format. Human annotators can infer certain details missing from the notes from experience or familiarity with the surgeon (eg, legacy knowledge). However, such familiarity is difficult to learn for the model in a data driven manner.

One factor that facilitated our efforts in comparing different versions was our extensive efforts in mapping data between registry versions into a common data model. This has enabled us to perform large retrospective studies through time.^30^ We recognize that not every institute has or can do such a mapping. Public resources such as Observational Medical Outcomes Partnership Common Data Model (OMOP CDM)^31^ or Unified Medical Language System (UMLS) Metatherasus^32^ are dictionaries which can be used to standardize data.

Automating registry abstraction is directly comparable to mapping clinical text to medical coding. The International Classification of Diseases (ICD)^33^ code is a long-established tool to process and classify diseases. Automation in registry abstraction is analogous to mapping diagnostic texts to ICD-10 codes with its similar text input and desired output labels. Recent work show how deep learning have provided not only monolingual but multilingual^34^ classifiers to render diagnostic texts into standardized ICD framework.^35^ Automation in registries has been developed in other subdomains in clinical setting such as central cancer registries^36^ and healthcare-associated infections.^37^

### Limitations

The scope of this work is limited to procedural text and variables. We recognize that much of the information required by the STS registry (eg, comorbidity information) is not expected to be included in surgical notes. Information such as comorbidity requires combing through lengthy patient documentation, where a single patient would not only have operative notes but other types of notes. Incorporating additional note types is warranted to provide even broader data extraction. Second, our classifier currently does not perform well in rare procedures such as MV prosthetic valve repair, trauma repair, and valve sparing root remodeling Yacoub. Accurate reporting of such procedures is particularly important for outcomes reporting and research. However, our work covers 95% of all cardiac procedures, therefore likely covering the most time-consuming manual abstraction.

## Conclusion

In this work, we show a PLM (CS-BERT) can accurately identify common cardiac surgical procedures from free-text operative notes. The model identifies more granular procedural details compared to CPT codes, is generalizable to unseen sites, and robust against semantic shift in data. Most importantly, automatically abstracted procedures from CS-BERT yielded similar aggregate outcomes compared to manually abstracted data. Such a model has the potential to automate parts of cardiac surgical outcomes reporting, thereby increasing the coverage of national registries to lower resourced sites by minimizing the need for specialized workforce dedicated to quality improvement.

## Supplementary Material

ooae054_Supplementary_Data

## Data Availability

The data used for this work cannot be shared publicly due to inclusion of protected health information data and need for a data use agreement with the institution.

## Code availability

The code used in this paper is available on Github: https://github.com/JaehyunsLee/sts_classifier.
