# Supplementary material for: Automating surgical procedure extraction for society of surgeons adult cardiac surgery registry using pretrained language models
Source: JAMIA Open. 2024 Jul 24;7(3):ooae054. doi: 10.1093/jamiaopen/ooae054 (PMC11268872; doi:10.1093/jamiaopen/ooae054)
Supplement: ooae054_Supplementary_Data [file ooae054_supplementary_data.zip › auto_procs_wAI_JAMIA_supplemental_v2.docx]

SUPPLEMENTAL MATERIAL

**STS-ACSD data mapping**

The format of the raw STS-ACSD data elements is often categorical with multiple options per data-element. These data elements may change through time, although the actual data may not change. For example, valve sparing aortic root surgery was classified under several different elements over time. We harmonized this data by mapping the individual data elements into a common data model which was then used for prediction. Each version of the STS-ACSD was saved into a separate SQL table. SQL functions were then used to map elements into a single consistent data table. This code to do the mapping, as well as details for each are shown in Supplemental Table 1.

[This table is included separately due to the size of the table]

Supplemental Table 1: Variable mapping between versions of STS-ACSD. Also includes detailed explanation of each variable.

[This table is included separately due to the size of the table]

Supplemental Table 2: Variable-by-variable breakdown of CS-BERT performance

Supplemental Figure 1: Fine-Tuning CS-BERT with only learning from scratch from the top K-layers within the top transformer block. The other remaining layers are duplicated from the original BERT (e.g., BioClinicalBERT) model.
