# Supplementary figures and images for: Automating surgical procedure extraction for society of surgeons adult cardiac surgery registry using pretrained language models
Source: JAMIA Open. 2024 Jul 24;7(3):ooae054. doi: 10.1093/jamiaopen/ooae054 (PMC11268872; doi:10.1093/jamiaopen/ooae054)

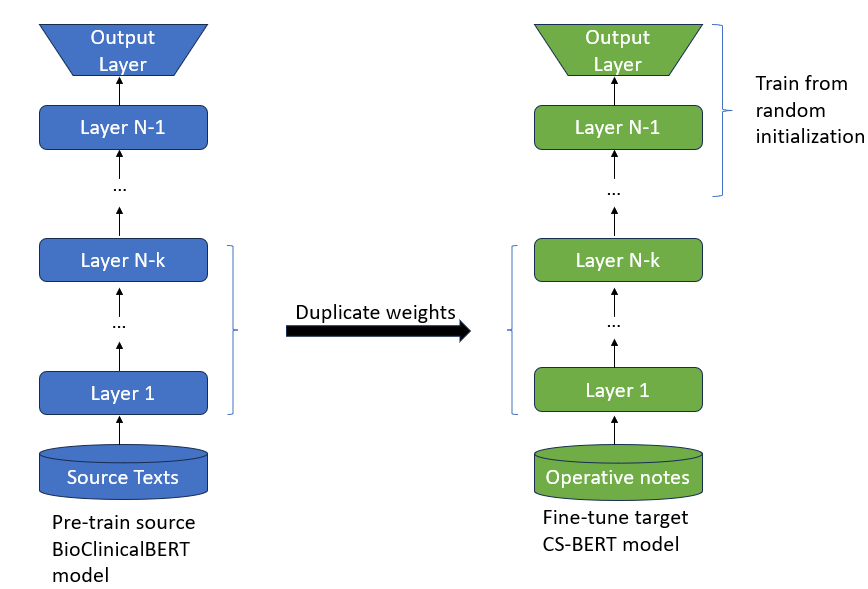

Supplement: ooae054_Supplementary_Data [file ooae054_supplementary_data.zip › supp_figure1.png]
